# Association Between BAK1 Gene rs210138 Polymorphisms and Testicular Germ Cell Tumors: A Systematic Review and Meta-Analysis

**DOI:** 10.3389/fendo.2020.00002

**Published:** 2020-01-23

**Authors:** Jiaxuan Qin, Yufeng Yang, Xuan Zhuang, Jinchun Xing

**Affiliations:** ^1^Department of Urology Surgery, The First Affiliated Hospital of Xiamen University, Xiamen, China; ^2^Center of Diagnosis and Treatment of Urinary System Diseases, The First Affiliated Hospital of Xiamen University, Xiamen, China; ^3^The Key Laboratory of Urinary Tract Tumors and Calculi of Xiamen City, The First Affiliated Hospital of Xiamen University, Xiamen, China

**Keywords:** BAK1, rs210138, single nucleotide polymorphisms, testicular germ cell tumors, meta-analysis

## Abstract

**Background:** Several studies including some genome-wide association studies (GWAS) had shown that BAK1 gene rs210138 polymorphisms might be associated with testicular germ cell tumors (TGCT). Here we tried to sum up the association through a systematic review and meta-analysis.

**Methods:** Studies associated with BAK1 rs210138 and TGCT was systematically searched in databases. The effect size was pooled according to ORs and 95% CIs.

**Results:** Our systematic review and meta-analysis comprised 14 articles. Significantly increased risk of TGCT was found in eligible GWAS and follow-up studies, in overall group and its Caucasian subgroup.

**Conclusions:** Compared with adenine (A), BAK1 rs210138 guanine (G) is associated with increased risk of TGCT. Well-planned studies with larger sample size and more subgroups are needed to verify the risk identified in our systematic review and meta-analysis.

## Background

More than 90% of cancers of the testicle develop in germ cells. Testicular germ cell tumor (TGCT) manly consist of seminomas and non-seminomas (1). The protein encoded by BCL2 antagonist/killer 1 (BAK1) gene belongs to the BCL2 protein family. BAK1 protein localizes to mitochondria, and functions to promote apoptosis. In a kind of Bak^(−/−)^ mice, 60% mice harbored high-grade tumors within the testis (2). Several studies including some genome-wide association studies (GWAS) (3) had shown that BAK1 gene rs210138 polymorphisms might be associated with TGCT. Here we tried to sum up the association through a systematic review and meta-analysis.

## Methods

### Identification of Eligible Studies

Independently, two researchers systematically searched these databases: GWAS Catalog, Wanfang, CNKI, clinicaltrials.gov, Cochrane Library, PubMed, Embase. Term “rs210138” was used in GWAS Catalog. And in other databases, these terms were used without limitation: “BAK1” AND “cancer of testis OR carcinoma of testis OR testicular cancer OR testis cancer OR ball cancer OR testicular germ cell tumors OR TGCT” AND “polymorphisms OR polymorphism.” The last search update was on Nov 30, 2018. For additional studies, we made a manual search in reviews and references of related studies.

### Inclusion and Exclusion Criteria

Independently, two researchers made the selection according to the following inclusion criteria: (1) evaluation of the association between BAK1 rs210138 and TGCT susceptibility; (2) case-control study; (3) studies focusing on tissues of human beings; (4) elaborate genotype data in non-GWAS study or enough data in GWAS study could be acquired. Exclusion criteria: (1) duplication of previous publications (When there were multiple publications from the same population, only the largest study was included); (2) review, comment, and editorial; (3) study without enough data; (4) studies focusing on cell lines. Dissertation thesis were included in the analysis. We took experience and inspiration from the article we have published recently (4), in which the inclusion and exclusion criteria was mature and rigorous.

### Data Extraction

Data of the eligible studies were extracted by two investigators independently. Conflict was solved by discussion. The third investigator would be involved if necessary. Try to get detailed genotype data by contacting the author.

The following contents were collected: year of publication, the characteristics of cases and controls, first author's surname, ethnicity, Hardy-Weinberg equilibrium, source of control groups, country of origin, genotyping method, number of cases, and controls.

### Methodological Quality Assessment

According to Newcastle-Ottawa Scale (NOS) (5), two investigators evaluated qualities of included studies independently. Quality scores range from 0 to 10, and higher scores means better quality of the study. The most important factor was “age, gender, and country.” Conflict was solved by discussion.

### Statistics Analysis

This meta-analysis complied with the PRISMA checklists (6). Pooled ORs and 95% CIs were calculated to evaluate the strength of the association between BAK1 gene rs210138 polymorphisms and TGCT susceptibility. Evaluation of Hardy-Weinberg equilibrium (HWE), OR and 95% CIs, heterogeneity, sensitivity, and publication bias were performed according to methods in our published article (4). In addition, I–V random effects model was used in total to get all data shown in Table 4 except overall subgroup. Sensitivity analyses and potential publication bias were not performed in Table 4. Stata 12.0 software (StataCorp, College Station, Texas, USA) were used in all statistical analyses. We regarded two-tailed *P* < 0.05 as significant except for specified conditions, where a certain *P*-value was declared.

## Results

### Characteristics of Studies

**Figure 1:**
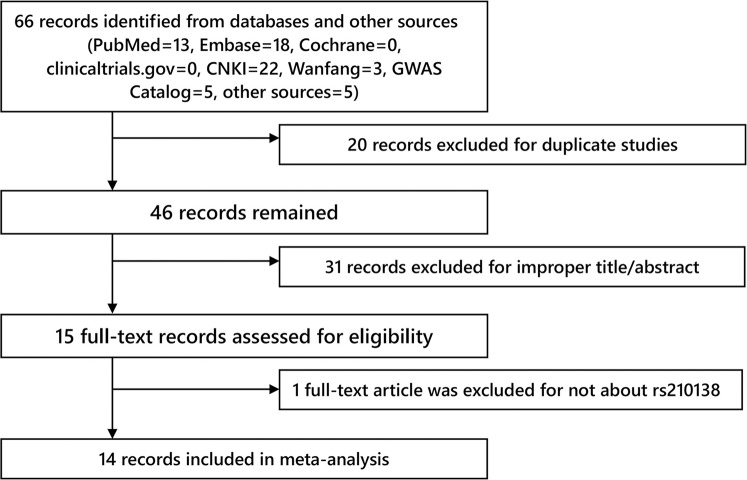
Flow chart of study selection.

In total, we captured 66 articles from databases (PubMed = 13, Embase = 18, Cochrane = 0, clinicaltrials.gov = 0, CNKI = 22, Wanfang = 3, GWAS Catalog = 5, other sources = 5). Figure 1 displayed the selection process. One full-text article was excluded for not about rs210138. Finally, 14 records (7–20) were included in our systematic review and meta-analysis. Tables 1, 2 displayed characteristics of each study. The control group of study Poynter et al. (7) and Dantsev et al. (10) had shown significant departure from HWE.

**Table 1:** Characteristics of studies about BAK1 rs210138 included in the systematic review and meta-analysis.

**No**.	**Study ID**	**Year**	**Country or area**	**Ethnicity**	**Control type**	**Genotyping method**	**Case**			**Control**			P for HWE^*^	**Quality**
	**With detailed genotype**						**GG**	**AG**	**AA**	**GG**	**AG**	**AA**		
1.1	Poynter et al.^*^ (7)	2012	USA	Caucasian	PB^*^	Sequencing	3	4	8	6	16	57	0.007^*^	6
1.2	**Lessel et al**. (8)	2012	Croatia	Caucasian	PB	TaqMan	30	109	179	14	87	221	0.156	8
1.3	**Duan** (9)	2016	China	Chinese	PB	Sequencing	16	31	29	12	63	73	0.756	7
1.3.1				Han			11	16	10	9	40	34	0.584	7
1.3.2				Uygur			5	15	19	3	23	39	0.867	7
1.4	**Dantsev et al**. (10)	2018	Russia	Caucasian	PB	PCR-RFLP	7	53	82	0	50	103	0.016^*^	9
	**Follow up (G vs. A)**						Case/Control, RA^*^, OR^*^ (95%CI^*^), ***P*****-value**							**Label**
2.1	Kratz et al.^*^ (11) (follow up)	2011	USA	Caucasian	PB	Illumina Custom iSelect bead chip	119/871, G, 1.80 (1.35–2.41), 7.03 × 10^∧^ (−5)							**FTCS**^**#**^
2.2	Marcotte et al.^*^ (12) (follow up)	2017	USA	Caucasian	NA	Illumina HumanCoreExome12	91 complete case-parent trios, G, 3.31 (1.89–5.79), NA^*^							**CCRN**^**#**^
	**GWAS (G vs. A)**						**Case/Control, RA, OR (95%CI)**, ***P*****-value**							**Label**
3.1	**Rapley et al**. (13)	2009	UK	Caucasian	PB									**UKTCC**^**#**^
3.1.1	Discovery study (GWAS)					Illumina 370K (cases) Illumina 550K (controls)	730/1,435, G, 1.50 (1.30–1.74), 4.5 × 10^∧^ (-8)							
3.1.2	Replication study (follow up)					TaqMan	571/1,806, G, 1.50 (1.28–1.75), 6.6 × 10^∧^ (−7)							
3.2	**Ruark et al**. (14)	2013	UK	Caucasian	PB									**UKTCC**
3.2.1	Discovery study (GWAS)					Illumina HumanCNV370Duo (cases) Illumina Infinium 1.2M (controls)	986/4,946, G, 1.55 (1.39–1.73), 1.47 × 10^∧^ (−14)							
3.2.2	Replication study (follow up)					custom Illumina Infinium array	1,064/10,082, G, 1.44 (1.30–1.61), 1.49 × 10^∧^ (−11)							
3.3	**UKTCC** **+** **Sweden/Norway** (15)						5,518/19,055, G, 1.48 (1.42–1.54), 2.9 × 10^∧^ (−37)							
3.3.1	Litchfield et al. (15) (GWAS)	2017	UK	Caucasian	PB	Oncoarray platform	3,206/7,422, G, 1.42 (1.35–1.49), 8.5 × 10^∧^ (−22)							**UKTCC**
3.3.2	Kristiansen et al. (16) (GWAS)	2015	Sweden/Norway	Caucasian	PB	NA (cases) Illumina OmniExpress (controls)	1,327/6,687, G, NA, NA							**Sweden/Norway**
3.2.1	Ruark et al. (14) (GWAS)	2013	UK	Caucasian	PB	as 3.2.1	986/4,946, G, 1.55 (1.39–1.73), 1.47 × 10^∧^ (−14)							**UKTCC**
3.4	**NCI** (17, 18) (GWAS)	NA	USA	Caucasian	PB	Illumina 660K	582/1,056, G, 1.62 (1.35–1.95), 2.83 × 10^∧^ (−7)							**NCI**^**#**^
3.4.1	STEED (GWAS)						479/555, G, NA, NA							**STEED**^**#**^
3.4.2	FTCS (GWAS)						103/501, G, NA, NA							**FTCS**^**#**^
3.5	**NCI** **+** **USC** (19)						940/1,559, G, 1.598 (1.38–1.85), 2.76 × 10^∧^ (−10)							
3.5.1	Schumacher et al. (19) (GWAS)	NA	USA	Caucasian	PB	Illumina 610K	358/503, G, NA, NA							**USC**^**#**^
3.4	NCI (17, 18) (GWAS)	NA	USA	Caucasian	PB	As 3.4	582/1,056, G, 1.62 (1.35–1.95), 2.83 × 10^∧^ (−7)							**NCI**
3.6	**Kanetsky et al**. (20)	2011	USA	Caucasian										**UPENN**^**#**^
3.6.1	Discovery study (GWAS)				HB^*^	Affymetrix 6.0	349/914, G, 1.34 (1.08–1.65), 0.0074							
3.6.2	Replication study (follow up)				PB	iPLEX Mass Array	397/862, G, 1.23 (0.99–1.52), 0.065							

* *HWE, Hardy–Weinberg equilibrium; PB, population-based; HB, hospital-based; RA, risk allele; OR, Odds ratio; CI, confidence interval; NA, not available*.

* *Results with statistical significant difference were marked as bold*.

* *Poynter et al.'s study focused on pediatric GCTs with age <22 years at diagnosis, and several extragonadal germ cell tumor male cases were included*.

* *Kratz et al.'s study used generalized estimating equations method to get OR and 95% CIs, and included 97 cases with familial TGCT and 22 cases with sporadic bilateral TGCT*.

* *Marcotte et al.'s study focused on pediatric GCTs with age <20 years at diagnosis, included 91 TGCT complete case-parent trios, and used transmission disequilibrium test method to get OR and 95% CIs*.

# *Detailed in Table 2*.

**Table 2:** Characteristics of cases and controls.

**Study ID**	**Case**	**Control**
Kratz et al. (11)	From NCI Clinical Genetics Branch Familial Testicular Germ Cell Study (FTCS).	From the Prostate, Lung, Colorectal, Ovarian (PLCO) Cancer Screening Trial.
Marcotte et al. (12)	From the Children's Oncology Group Childhood Cancer Research Network (CCRN).	Parents of cases.
Rapley et al. (13)	From a UK study of familial testicular cancer and a national collection of TGCT Cases treated within the UK. Cases were recruited via the UK Testicular Cancer Collaboration (UKTCC).	From the 1958 Birth Cohort (1958BC).
Ruark et al. (14)	From a UK study of familial testicular cancer and a national collection of TGCT Cases treated within the UK. Cases were recruited via the UK Testicular Cancer Collaboration (UKTCC).	Controls for the GWAS: 2,482 from the 1958 Birth Cohort (1958BC) and 2,587 from the UK National Blood Service (NBS). Controls for the iCOGs replication: 814 age <65 male from a study of Prostate Cancer (UKGPCS), 7,871 controls (6,627 female and 1,244 male) from a SEARCH (Study of Epidemiology & Risk Factors in Cancer), 1,397 females from the BBCS (British Breast Cancer Study).
Litchfield et al. (15)	From a UK study of familial testicular cancer and a national collection of TGCT Cases treated within the UK. Cases were recruited via the UK Testicular Cancer Collaboration (UKTCC).	2,976 male from the UK Genetic Prostate Cancer Study (UKGPCS) (age <65) and SEARCH (Study of Epidemiology & Risk Factors in Cancer), 4,446 female from Breast Cancer Association Consortium (BCAC).
Kristiansen et al. (16)	Recruitment of Norwegian TGCT patients diagnosed between 1990 and 2008 was based on data from the Cancer Registry of Norway. Recruitment of Swedish TGCT patients diagnosed between 1995 and 2006 was based on data from the Swedish National Cancer Registry.	From the TwinGene project, conducted between 2004 and 2008, is a population-based Swedish study of twins born between 1911 and 1958.
NCI (17, 18)	From NCI Clinical Genetics Branch Familial Testicular Germ Cell Study (FTCS) and US Servicemen's Testicular Tumor Environmental and Endocrine Determinants Study (STEED).	From the Prostate, Lung, Colorectal, Ovarian (PLCO) Cancer Screening Trial and STEED.
USC (19)	Individuals analyzed are part of a population-based study at the University of Southern California (USC) based in the California and the California Cancer Registry (CCR).	Controls from the NCI Breast & Prostate Cancer Cohort Consortium genome-wide study of aggressive prostate cancer.
Kanetsky et al. (20)	Most cases of discovery study were from the University of Pennsylvania (UPENN) Health System or Fox Chase Cancer Center. Cases of replication study were from western Washington State.	Controls of discovery study were from the University of Pennsylvania Catheterization Study (PennCATH). Controls of replication study were from western Washington State.

### Meta-Analysis Overall

**Figure 2:**
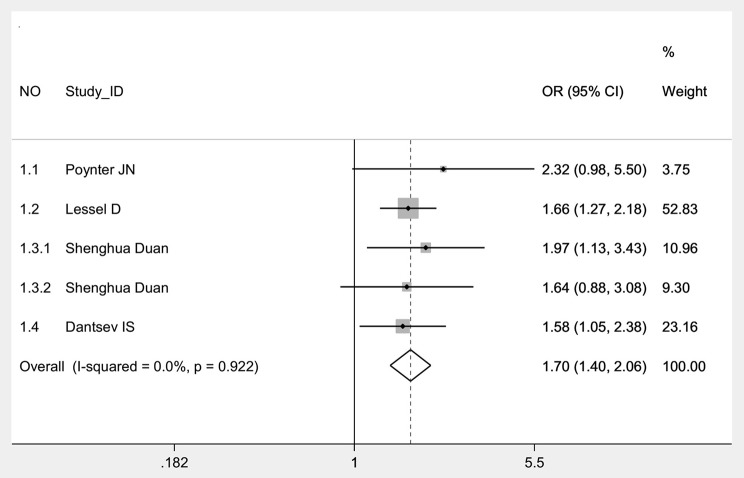
Forest plot with a fixed effects model for the association between BAK1 rs210138 and TGCT in allelic comparison (G vs. A) overall. For each study, the estimate of OR and its 95% CI is plotted with a box and a horizontal line. Rhombus: pooled OR and its 95% CI.

In overall group and its Caucasian subgroup, significantly increased risk of TGCT was found in all genetic models of BAK1 rs210138 (Table 3 and Figure 2). And the results showed stability in sensitivity analyses in four genetic models (Table 3). In heterozygote comparison (AG vs. AA), when study NO 1.2 was excluded, statistically different results were obtained (Table 3). No significant publication bias was found in Egger's test or Begg's test in either genetic models of overall group. Publication bias was not performed in Caucasian subgroup because of scanty data.

**Table 3:** Summary of pooled ORs in the meta-analysis with detailed genotype.

**BAK1 rs210138**	**Number (cases/ controls)**	**G vs. A**		**GG vs. AA**		**AG vs. AA**		**AG** **+** **GG vs. AA**		**GG vs. AA** **+** **AG**	
		OR^*^ (95%CI^*^)	***I*^**2**^ (%)**	**OR (95%CI)**	***I*^**2**^ (%)**	**OR (95%CI)**	***I*^**2**^ (%)**	**OR (95%CI)**	***I*^**2**^ (%)**	**OR (95%CI)**	***I*^**2**^ (%)**
Overall	551/702	1.701 (1.403–2.062)^*^	0.0	**3.424 (2.085–5.622)**	0.0	1.459 (1.136–1.876)^*^	0.0	**1.671 (1.318–2.119)**	0.0	**2.974 (1.848–4.786)**	0.0
Caucasian subgroup	475/554	**1.671 (1.345–2.076)**	0.0	**3.266 (1.810–5.891)**	0.0	***1.482 (1.127–1.949)***	0.0	**1.663 (1.282–2.156)**	0.0	**2.830 (1.585–5.052)**	0.0

* *OR, odds ratio; CI, confidence interval*.

* *Results with statistical significant difference were marked as bold. Unstable results in sensitivity analyses were marked as italic*.

### Meta-Analysis in Total

**Figure 3:**
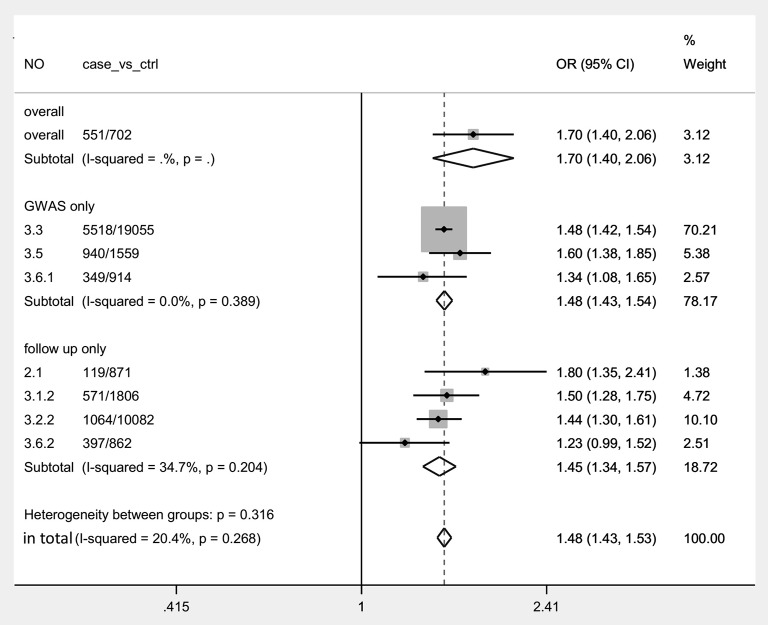
Forest plot with an I–V random effects model for the association between BAK1 rs210138 and TGCT in allelic comparison (G vs. A) in total. For each study, the estimate of OR and its 95% CI is plotted with a box and a horizontal line. Rhombus: pooled OR and its 95% CI.

In our eligible GWAS and follow-up studies, we could not retrieve elaborate genotype data. Without elaborate genotype data, sensitivity analyses and potential publication bias were not performed. We tried to perform a meta-analysis based on OR and 95% CIs by using Stata 12.0 software in allelic comparison (G vs. A) (Figure 3), and I–V random effects model was used to get all data shown in Table 4 except overall group. Significantly increased risk of TGCT was found in all groups in Table 4.

**Table 4:** Summary of pooled ORs in the meta-analysis in total.

**BAK1 rs210138**	**Number (cases/controls)**	**G vs. A**		**No of studies included**
		OR^*^ (95%CI^*^)	***I*^**2**^ (%)**	
Overall	551/702	1.701 (1.403–2.062)^*^	0.0	1.1; 1.2; 1.3.1; 1.3.2; 1.4
GWAS only	6,807/21,528	**1.483 (1.427–1.541)**	0.0	3.3; 3.5; 3.6.1
Follow up only	2,151/13,621	**1.448 (1.339–1.566)**	34.7	2.1; 3.1.2; 3.2.2; 3.6.2
GWAS + follow up	8,958/35,149	**1.476 (1.426–1.528)**	11.4	GWAS only + follow up only
In total	9,509/35,851	**1.483 (1.433–1.534)**	20.4	Overall + (GWAS + follow up)

* *OR, odds ratio; CI, confidence interval*.

* *Results with statistical significant difference were marked as bold*.

## Discussion

Above all, we found BAK1 rs210138 guanine (G) was associated with increased risk of TGCT in most genetic models in the meta-analysis of single case-control studies. In GWAS studies, follow-up studies and their meta-analysis based on OR and 95% CIs, BAK1 rs210138 guanine (G) also showed association with increased risk of TGCT in allelic comparison, which was consistent with the results in the meta-analysis of single case-control studies.

Meanwhile, our meta-analysis had several limitations which should be mentioned. Up to now, number of eligible studies for our meta-analysis were small. There was inadequate data for subgroup analyses. Omission of studies in other languages or unpublished studies might happened. In our eligible GWAS and follow-up studies, we could not retrieve elaborate genotype data. With those limitations, the study provided some insights on the potential association between BAK1 rs210138 and TGCT susceptibility.

## Conclusion

Our results suggested that: Compared with adenine (A), BAK1 rs210138 guanine (G) is associated with increased risk of TGCT. Well-planned studies with larger sample size and more subgroups are needed to verify the risk identified in our systematic review and meta-analysis.

## Ethics Statement

The Ethics Committee of the First Affiliated Hospital of Xiamen University approved the study protocol. Written informed consent was obtained from all patients enrolled in the investigation.

## Author Contributions

JQ and JX designed the study, drafted and substantively revised the manuscript, and proof the English language. JQ, YY, and XZ accumulated the data. JQ and YY were for analysis and interpretation of the data. All authors read and approved the final manuscript.

### Conflict of Interest

The authors declare that the research was conducted in the absence of any commercial or financial relationships that could be construed as a potential conflict of interest.

## Abbreviations

CNKI: China National Knowledge Infrastructure
CI: confidence intervals
GWAS: genome-wide association studies
HWE: Hardy-Weinberg equilibrium
NOS: Newcastle-Ottawa Scale
OR: odds ratio
TGCT: testicular germ cell tumors.
